# Molecular evidence of high-risk human papillomavirus infection in colorectal tumours from Cuban patients

**DOI:** 10.1590/0074-02760160217

**Published:** 2016-10-31

**Authors:** Yudira Soto, Celia Maria Limia, Licet González, Bienvenido Grá, Olga Marina Hano, Pedro Ariel Martínez, Vivian Kourí

**Affiliations:** 1 Institute of Tropical Medicine Pedro Kourí, Department of Virology, Laboratory of Sexually Transmitted Diseases,La Habana, Cuba; 2 Institute of Gastroenterology, La Habana, Cuba

**Keywords:** human papillomavirus, colorectal cancer, real-time PCR

## Abstract

The association between colorectal cancer and human papillomavirus (HPV) infection is still unproven. The aim of this study was to investigate the presence of high-risk HPV (HR-HPV) DNA in colorectal tissues from Cuban patients. A total of 63 colorectal formalin-fixed paraffin-embedded tissues were studied (24 adenocarcinoma, 18 adenoma, and 21 colorectal tissues classified as benign colitis). DNA from colorectal samples was analysed by quantitative real-time polymerase chain reaction to detect the most clinically relevant high HR-HPV types (HPV-16, -18, -31, -33, -45, -52, and -58). Associations between histologic findings and other risk factors were also analysed. Overall, HPV DNA was detected in 23.8% (15/63) of the samples studied. Viral infections were detected in 41.7% of adenocarcinoma (10/24) and 27.7% of adenoma cases (5/18). HPV DNA was not found in any of the negative cases. An association between histological diagnosis of adenocarcinoma and HPV infection was observed (odd ratio = 4.85, 95% confidence interval = 1.40-16.80, p = 0.009). The only genotypes identified were HPV 16 and 33. Viral loads were higher in adenocarcinoma, and these cases were associated with HPV 16. This study provides molecular evidence of HR-HPV infection in colorectal adenocarcinoma tissues from Cuban patients.

Infection with a high-risk human papillomavirus (HR-HPV) type is necessary for the development of cervical carcinoma (Muñoz 2000). Among these, types 16 and 18 are the most common found in cervical cancer (CC) tumours (Muñoz et al. 2003); they are associated with approximately 76% of CC and 80% of anal cancers (Smith et al. 2007, de Vuyst et al. 2009). In total, 5.2% of the worldwide cancer burden is attributable to HPV (Yue et al. 2013). HPV DNA has been detected in tumour tissues of patients with head and neck cancers (Ringstrom et al. 2002), oral cancer (Ritchie et al. 2003), oesophageal cancer (Dai et al. 2007), some skin cancers (Connolly et al. 2014), and lung cancer (Sagerup et al. 2014, Zhai et al. 2015). Over the last few years, a possible correlation between HPV infection and colorectal cancer (CRC) has been suggested, based on several researchers detecting HPV DNA in these cancers, using different techniques (Buyru et al. 2006, Damin et al. 2007, Pérez et al. 2010, Chen et al. 2012).

CRC is the fourth most common cancer worldwide. It is estimated to be responsible for approximately 1,360,602 new cases and 693,881 deaths annually (Darragh & Winkler 2011). Approximately 95% of CRC are adenocarcinomas, with adenomas as the main precursor lesions (van der Loeff et al. 2014). The progression of adenoma to adenocarcinoma is inﬂuenced by environmental and lifestyle factors, sequential genetic changes, and possibly infections (Burnett-Hartman et al. 2013).

Findings of the present study indicate the presence of HPV DNA in colorectal tumours and suggest a role for oncogenic HPV types in the establishment of colorectal adenocarcinomas. These results may contribute to an understanding of the aetiology of sporadic CRC.

To explore the possible relationship between HPV infection and colorectal carcinogenesis, we investigated the presence of HPV DNA in CRC tissues from Cuban patients and examined the relationship between HR-HPV genotypes and viral loads and adenocarcinomas.

## MATERIALS AND METHODS

*Study population* - For this purpose, a cross-sectional study to detect HPV infection in colorectal tissues was performed. Study participants were outpatients at the Cuban National Institute of Gastroenterology between April and August 2014. Patients contributed only one sample for viral detection. The inclusion criteria were no prior surgery, radiation, or cytotoxic therapy for colorectal adenocarcinoma. Diagnoses of samples from colorectal adenomas, adenocarcinomas, and tissues with unremarkable pathological changes (a non-malignant control group) were confirmed by pathologists through standard criteria. In the study, 63 colorectal paraffin-embedded tissues were included: 42 from patients with CRC and 21 from individuals without CRC. Of the 42 tumours sampled, 24 were adenocarcinomas and 18 adenomas. Twenty-one specimens diagnosed as chronic inflammatory infiltrates of colorectal mucosa due to benign colitis were used as the non-malignant control group.

Colorectal tissues were obtained from the rectum, rectosigmoid junction, and sigmoid colon. Complete clinical and pathological data were recorded, including patients’ clinical histories and demographics (age at the time of sampling, gender, skin colour), family antecedents, grade of tumour differentiation, and tumour location.

*DNA extraction* - *HPV detection and genotyping* - Formalin-fixed paraffin-embedded (FFPE) colorectal tissues were obtained. Between seven and 10 slides of approximately 5-µM wide sections of tissue were deparaffinised in xylene and absolute ethanol, and then DNA was obtained using a QIAamp DNA FFPE Tissue Kit (Qiagen, Germany), following the manufacturer’s protocol. The potential for sample-to-sample cross-contamination was limited by performing DNA extractions from only six tissue samples per day. DNA quality was evaluated by polymerase chain reaction (PCR) amplification of a fragment from the human β-globin gene using primers PC04/GH20 (Gravitt et al. 2000).

Quantitative real-time PCR (qRT-PCR) was performed as previously described (Schmitz et al. 2009, Soto et al. 2012) to identify the most frequent genotype of HR-HPV and quantify viral loads. PCR primers and corresponding TaqMan probes were used to amplify the LCR/E6/E7 region of HPV-16, -18, -31, -33, -45, -52, and -58 genomes (Schmitz et al. 2009, Soto et al. 2012).

HPV 16 and 18 standard curves were constructed from purified genomic DNA from HPV cultures in SiHa and HeLa cell lines, respectively. The curves showed good linear correlation (r = 0,99) and low error values throughout the range of the six target DNA concentrations. The assay had a detection limit of 10 copies for HPV DNA for all genotypes tested. The detection limit for HPV18 and 45 was also of 10 copies of viral DNA. No cross reactions between HPV and other DNA viruses were observed (Schmitz et al. 2009, Soto et al. 2012).

To identify HR-HPV types and quantify viral loads, single PCR reactions were performed for each HPV type. To control for DNA quality, β-globin was amplified in each sample. PCR was performed using a LightCycler 1.5 platform (Roche Diagnostics, Indianapolis, USA). The PCR reaction mixture comprised 5 µL of DNA (up to 50 ng), 4 µL of Quantitative PCR TaqMan Master Mix (Roche Diagnostics), 10 pmol of each primer, and 1-5 pmol of each probe in a final volume of 25 µL. The initial denaturation step at 94ºC for 10 min was followed by 45 PCR cycles at 94ºC for 15 s, 50ºC for 20 s, and 60ºC for 40 s each.

*Statistical analysis* - Epidemiological and clinical data were collected in one-on-one interviews in a private room, using a standardised questionnaire. Data were processed using IBM SPSS Statistics 20. Proportions were compared by Chi-squared testing. Odds ratios (ORs) and 95% confidence intervals (CIs) were calculated in a univariate logistic regression to estimate the relative risk of HPV detection associated with different variables, including age at the time of sampling, gender, skin colour, family antecedents, grade of tumour differentiation, and tumour location. Comparisons between viral loads from groups of patients with different histological classifications or HPV genotypes were made using Kruskall-Wallis and Mann-Whitney tests. All statistical tests were considered to be significant at a p-value < 0.05.

*Ethics* - This study was approved by the Ethics Committee of the Institute of Tropical Medicine Pedro Kourí and complies with the principles established in the Helsinki Declaration of 1975 and revised in 1983 (WMA 2008). Written informed consent was obtained from each study participant before the interview, sample collection, and testing.

## RESULTS

Overall, HPV DNA was detected in 23.8% (15/63) of the samples tested. HPV infection was detected in 41.7% of adenocarcinoma (10/24) and 27.7% of adenoma cases (5/18), but was not detected in any of the negative cases (p = 0.002). An association between histological diagnosis of adenocarcinoma and HPV infection was observed (OR = 4.85, 95% CI = 1.40-16.80, p = 0.009). HPV infections were exclusively detected in the rectum (60%, 9/15) and in rectosigmoid junction (40%, 6/15) of colorectal samples (Table I).

**TABLE I:** Analysis of epidemiological data and histology, and their association with human papillomavirus (HPV) infection

Variables		Total (N = 63) n (%)	HPV positive (N = 15) % (n/N)	HPV negative (N = 48) % (n/N)	p-value	OR (CI 95%)
Age (years) n = 63	≤ 40 41-50 51-60 > 60	7 (11.1) 12 (19.1) 13 (20.6) 31 (49.2)	6.7 (1/15) 26.7 (4/15) 20 (3/15) 46.6 (7/15)	12.5 (6/48) 16.7 (8/48) 20.8 (10/48) 50 (24/48)	1.000 0.457 1.000 0.472	0.50 (0.05-4.52) 1.81 (0.46-7.17) 0.95 (022-4.02) 0.80 (0,25-2.57)
Sex n = 63	F M	31 (49.2) 32 (50.8)	66.7 (10/15) 33.3 (5/15)	43.8 (21/48) 56.2 (27/48)	0.120	2.57 (0.76-8.67)
Color of the skin n = 61	Black Mulatto White	41(67.2) 7 (11.5) 13 (21.3)	57.1 (8/14) 7.2 (1/14) 35.7 (5/14)	70.2 (33/47) 12.8 (6/47) 17.0 (8/47)	1.000 0.580 0.710	1.13 (0.20-6.39) 0.75 (0.64-0.87) 1.83 (0.35-9.47)
Histology results n = 63	Negative Adenoma Adenocarcinoma	21 (33.3) 18 (28.6) 24 (38.1)	0 (0/15) 33.3 (5/15) 66.7 (10/15)	43.8 (21/48) 27.1 (13/48) 29.1 (14/48)	0.002 0.746 0.009	- 1.34 (0.38-4.69) 4.85 (1.40-16.09)
Colorectal tissue location n = 63	Rectum Rectum-sigmoid Sigmoid	34 (54) 18 (28.6) 11 (17.4)	60 (9/15) 40 (6/15) 0	52.1 (25/48) 25 (12/48) 22.9 (11/48)	0.591 0.330 0.053	1.38 (0.42-4.48) 2.00 (0.58-6,79) -
Tumor differentiation n=23	Well Moderate	8 (34.8) 15 (65.2)	44.4 (4/9) 55.6 (5/9)	28.6 (4/14) 71.4 (10/14)	0.403	0.40 (0.07-2.18)
Family antecedents n = 63	Yes	11 (17.5)	20 (3/15)	16.7 (8/48)	0.714	1.25(0.28-5.46)

CI: confidence interval; OR: odd ratio.

Among HPV-positive cases, 66.7% had adenocarcinomas (10/15) and 33.3% had adenomas (5/15) (Table II). The only genotypes identified were HPV 16 and 33. HPV 16 was detected in 70% (7/10) of HPV-positive adenocarcinomas and in 100% of HPV-positive adenomas (5/5). HPV 33 infection was only identified in adenocarcinoma cases (60%, 6/10). Dual infections with HPV 16 and 33 were identified in 30% (3/10) of HPV-positive adenocarcinomas. Adenocarcinomas were significantly associated with HPV 33 infection (p = 0.002). Other variables such as tumour differentiation or anatomical location of the colorectal lesion were not found to be associated with a specific HPV type (Table III).

**TABLE II:** Comparison of age groups with histological results and human papillomavirus (HPV) infection

Age group N = 63	Negative for tumor N = 21			Adenomas N = 18			Adenocarcinomas N = 24		
	HPV positive n = 0	HPV negative n = 21	Total n (%)	HPV positive n = 5	HPV negative n = 13	Total n (%)	HPV positive n = 10	HPV negative n = 14	Total n = 24
< 40 (n = 7)	0	3	3 (14.3)	0	2	2 (11.1)	1	1	2 (8.3)
41-50 (n = 12)	0	7	7 (33.3)	1	1	2 (11.1)	3	0	3 (12.5)
51-60 (n = 13)	0	5	5 (23.8)	2	2	4 (22.2)	1	3	4 (16.7)
> 60 (n = 31)	0	6	6 (28.6)	2	8	10 (55.6)	5	10	15 (62.5)

**TABLE III:** Human papillomavirus (HPV) 16 and 33 infections and colorectal lesion characteristics

Variables		HPV 16 positive N = 12	p-value	HPV 33 positive N = 6	p-value
Histology results n = 63	Adenoma Adenocarcinoma	5 (41.7%) 7 (58.3%)	0.299 0.185	0 (0%) 6 (100%)	0.170 0.002
Colorectal tissue n = 63	Rectum Rectum-sigmoid Sigmoid	8 (66.7) 4 (33.3) 0 (0%)	0.358 0.729 0.104	3 (50%) 3 (50%) 0 (0%)	1.000 0.341 0.579
Adenocarcinoma differentiation n = 23	Well Moderate	3 (25%) 4 (33.3%)	0.647	1 (16.7%) 5 (83.36%)	0.621
Family antecedents n = 63	Yes	3 (25%)	0.425	0 (0%)	0.579

Viral loads were analysed and compare between the different histological classifications, adenomas and adenocarcinomas (Figure). The median viral loads were similar in adenocarcinoma and adenoma cases. The maximum viral load in adenomas was lower than that in adenocarcinomas (2.5 × 10^4^ copies/µL versus 3 × 10^6^ copies/µL, respectively). HPV 16 viral loads were higher than those of HPV 33 (3 × 10^6^ copies/µL versus 2.5 × 10^4^ copies/µL, respectively) (Figure).

**Figure:**
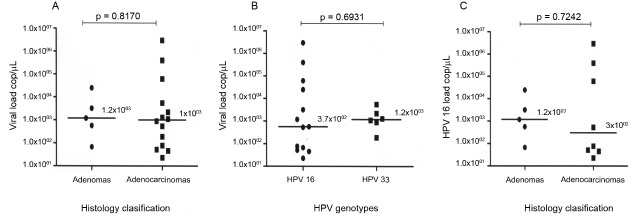
Comparison of the median (A) human papillomavirus (HPV) viral loads according to histological classification and (B) HPV 16 and 33 viral loads in colorectal tissues. (C) HPV 16 viral load according to histological classification.

Table I shows the relationship between HPV infection and variables analysed in the study population. HPV infection was more frequent in black individuals (57.1%, 8/14), in females (66.7%, 10/15), and in those over 60 years of age (46.6%, 7/15).

In the present study, the prevalence of HPV infection was higher in the group of patients over 60 years of age than in other age groups. However, no statistical association was found (p < 0.05) between HPV infection and age when proportions were compared by Chi-squared test (Table I).

The majority of patients with both colorectal lesions, adenomas and adenocarcinomas, were over 60 years of age (55.6% [10/18] and 62.5% [15/24], respectively). Furthermore, among patients with colorectal adenocarcinomas, HPV infection was more frequent in those over 60 years (33.3%, 5/15) (Table II).

Family antecedents of familial adenomatous polyposis were associated with the presence of adenomas (OR = 6.52, 95% CI = 1.61-26.38, p = 0.009).

## DISCUSSION AND CONCLUSIONS

Our study showed that oncogenic HPV types can be detected in colorectal adenocarcinoma tissues from Cuban patients. The absence of HPV DNA in non-malignant tissues suggests that the presence of HPV is not merely coincidental in colorectal carcinomas, but that it is a possible cofactor in development of the disease. In our study, an association between a histological diagnosis of adenocarcinoma and HPV infection was observed. Damin et al. (2013) published a meta-analysis of case-control studies that indicated a high prevalence of colorectal HPV infection in patients with CRC, as well as a significant increase in CRC risk associated with the presence of the virus.

Recently, Baandrup et al. (2014) conducted a meta-analysis of 37 studies. Of 2630 adenocarcinoma cases included, the pooled HPV prevalence was 11.2% (95% CI = 4.9-19.6%). The meta-analysis conducted by Baandrup et al. (2014) showed that studies in which five or more HPV were tested and five or more adenocarcinoma cases were HPV positive, the prevalence of HPV 16 in HPV positive adenocarcinoma cases ranged widely, from 18.2-85.7%. Applying the same criteria, the prevalence of HPV 33 ranged widely also; from 0-85.7%. According to this meta-analysis, both genotypes were the most frequently detected in adenocarcinoma.

A few studies have also investigated the presence of HPV in premalignant adenomatous polyps (adenomas). Data on the relationship between HPV infection and other factors, including genetic and molecular changes, and the development of CRC from adenomas are not conclusive. Cheng et al. (1995) found HPV DNA in 28% of 109 paraffin-embedded adenomas. In contrast, Burnett-Hartman et al. (2013) did not find HPV DNA in an assessment of 167 colorectal adenomas, 87 hyperplasic polyps, and 250 polyp-free controls.

According to our results, HPV infection was more frequent in females. Baandrup et al. (2014) also reported on the sex-specific HPV prevalences in colorectal adenocarcinomas. In two studies analysed, a non-significant tendency toward a higher HPV prevalence in women than men was found. In the remaining studies, the HPV prevalence in colorectal adenocarcinoma cases was similar between women and men.

In the present study, the prevalence of HPV infection was higher in patients over 60 years of age than in other age groups. However, no statistical association was found (p < 0.05). Furthermore, in the group of patients over 60 years of age, a higher proportion of HPV-positive cases had a histologic diagnosis of colorectal adenocarcinoma. According to demographic data analysis by Ranjbar et al. (2014), more than 90% of individuals with both CRC and HPV infections are older than 50 years of age. Our study shows an epidemiological pattern of CRC and HPV infection in a Cuban population that is similar to what was found in other studies (Damin et al. 2013, Ranjbar et al. 2014).

In the present study, family antecedents of familial adenomatous polyposis were associated with the presence of adenomas. Approximately 95% of CRCs are believed to have evolved from adenomatous polyps (adenomas). Various genetic and molecular changes occur as these polyps transform from benign to malignant. Familial adenomatous polyposis is a Mendelian dominant syndrome with an incidence of 1:11,000 that is caused by an alteration in the APC gene. The syndrome is characterised by the presence of adenomatous polyps in the gastroenteric tract, mostly in the colorectal-junction and duodenum, with a demonstrated adenoma-carcinoma sequence (Bronzino et al. 2003). There is no evidence to associate HPV infection with familial adenomatous polyposis (Toru & Bilezikci 2012).

In our study, HPV infections were exclusively detected in the rectum and rectosigmoid junction, suggesting that HPV infection may be a result of retrograde viral transmission from the anogenital area. However, this result differs from those of most previously published research, which have shown no relationship between HPV infection and the anatomic location of tumours. In these studies, rates of HPV detection in tissues collected from the rectum or sigmoid colon were similar to those in tissues obtained from the cecum or ascending colon. Because HPV infection is mainly transmitted by cell surface contact, the route of viral transmission to the colon remains to be determined (Chen et al. 2012, Ranjbar et al. 2014).

According to Damin et al. (2013), oncogenic HPV types may be associated with development of CRC. However, it should be noted that the detection of HPV DNA alone is not sufficient to establish a role for HPV in colorectal carcinogenesis.

Although further studies are needed to reach a definitive conclusion on the involvement of oncogenic HPV types in the establishment of colon adenocarcinomas, our study provides data that suggests a potential role for HR-HPV infection in the pathogenesis of CRC. Establishing a link between HPV infection and colorectal carcinogenesis, in addition to contributing to our understanding of the pathogenesis of this disease, could have important implications for patient management and prevention of CRC. If infection with HPV is found to have a causal role in colorectal carcinogenesis, then prophylactic vaccines against HPV could be used to prevent a large proportion of sporadic CRC.

## References

[B1] Baandrup L, Thomsen LT, Olesen TB, Andersen KK, Norrild B, Kjaer SK (2014). The prevalence of human papillomavirus in colorectal adenomas and
adenocarcinomas: a systematic review and meta-analysis. Eur J Cancer.

[B2] Bronzino P, Rassu PC, Cassinelli G, Stanizzi T, Casaccia M (2003). Familial adenomatous polyposis: review of the literature and report of
3 cases. G Chir.

[B3] Burnett-Hartman AN, Feng Q, Popov V, Kalidindi A, Newcomb PA (2013). Human papillomavirus DNA is rarely detected in colorectal carcinomas
and not associated with microsatellite instability: the Seattle colon cancer
family registry. Cancer Epidemiol Biomarkers Prev.

[B4] Buyru N, Tezol A, Dalay N (2006). Coexistence of K-ras mutations and HPV infection in colon
cancer. BMC Cancer.

[B5] Chen TH, Huang CC, Yeh KT, Chang SH, Chang SW, Sung WW (2012). Human papilloma virus 16 E6 oncoprotein associated with p53
inactivation in colorectal cancer. World J Gastroenterol.

[B6] Cheng JY, Sheu LF, Lin JC, Meng CL (1995). Detection of human papillomavirus DNA in colorectal
adenomas. Arch Surg.

[B7] Connolly K, Manders P, Earls P, Epstein RJ (2014). Papillomavirus-associated squamous skin cancers following transplant
immunosuppression: one Notch closer to control. Cancer Treat Rev.

[B8] Dai M, Zhang WD, Clifford GM, Gheit T, He BC, Michael KM (2007). Human papillomavirus infection among 100 oesophageal cancer cases in
the People’s Republic of China. Int J Cancer.

[B9] Damin DC, Caetano MB, Rosito MA, Schwartsmann G, Damin AS, Frazzon AP (2007). Evidence for an association of human papillomavirus infection and
colorectal cancer. Eur J Surg Oncol.

[B10] Damin DC, Ziegelmann PK, Damin AP (2013). Human papillomavirus infection and colorectal cancer risk: a
meta-analysis. Colorectal Dis.

[B11] Darragh TM, Winkler B (2011). Anal cancer and cervical cancer screening: key
differences. Cancer Cytopathol.

[B12] de Vuyst H, Clifford GM, Nascimento MC, Madeleine MM, Franceschi S (2009). Prevalence and type distribution of human papillomavirus in carcinoma
and intraepithelial neoplasia of the vulva, vagina and anus: a
meta-analysis. Int J Cancer.

[B13] Gravitt PE, Peyton CL, Alessi TQ, Wheeler CM, Coutlee F, Hildesheim A (2000). Improved amplification of genital human
papillomaviruses. J Clin Microbiol.

[B14] Muñoz N, Bosch FX, Sanjosé S de, Herrero R, Castellsague X, Shah KV (2003). Epidemiologic classification of human papillomavirus types associated
with cervical cancer. N Engl J Med.

[B15] Muñoz N (2000). Human papillomavirus and cancer: the epidemiological
evidence. J Clin Virol.

[B16] Pérez LO, Barbisan G, Ottino A, Pianzola H, Golijow CD (2010). Human papillomavirus DNA and oncogene alterations in colorectal
tumors. Pathol Oncol Res.

[B17] Ranjbar R, Saberfar E, Shamsaie A, Ghasemian E (2014). The aetiological role of human papillomavirus in colorectal carcinoma:
an Iranian population - based case control study. Asian Pac J Cancer Prev.

[B18] Ringstrom E, Peters E, Hasegawa M, Posner M, Liu M, Kelsey KT (2002). Human papillomavirus type 16 and squamous cell carcinoma of the head
and neck. Clin Cancer Res.

[B19] Ritchie JM, Smith EM, Summersgill KF, Hoffman HT, Wang D, Klussmann JP (2003). Human papillomavirus infection as a prognostic factor in carcinomas of
the oral cavity and oropharynx. Int J Cancer.

[B20] Sagerup CM, Nymoen DA, Halvorsen AR, Lund-Iversen M, Helland A, Brustugun OT (2014). Human papilloma virus detection and typing in 334 lung cancer
patients. Acta Oncol.

[B21] Schmitz M, Scheungraber C, Herrmann J, Teller K, Gajda M, Runnebaum IB (2009). Quantitative multiplex PCR assay for the detection of the seven
clinically most relevant high-risk HPV types. J Clin Virol.

[B22] Smith JS, Lindsay L, Hoots B, Keys J, Franceschi S, Winer R (2007). Human papillomavirus type distribution in invasive cervical cancer and
high-grade cervical lesions: a meta-analysis update. Int J Cancer.

[B23] Soto Y, Kourí V, Martínez PA, Correa C, Torres G, Goicolea A (2012). Standardization of a real-time based polymerase chain reaction system
for the quantification of human papillomavirus of high oncogenic
risk. Vaccimonitor.

[B24] Toru S, Bilezikci B (2012). Early changes in carcinogenesis of colorectal adenomas. West Indian Med J.

[B25] van der Loeff MFS, Mooij SH, Richel O, Vries HJ de, Prins JM (2014). HPV and anal cancer in HIV-infected individuals: A
Review. Curr HIV/AIDS Rep.

[B26] WMA - Wold-Medical-Association (2008). Declaration of Helsinki. Ethical principles for medical research involving
human subjects.

[B27] Yue Y, Yang H, Wu K, Yang L, Chen J, Huang X (2013). Genetic variability in L1 and L2 genes of HPV-16 and HPV-58 in
Southwest China. PLoS ONE.

[B28] Zhai K, Ding J, Shi HZ (2015). HPV and lung cancer risk: a meta-analysis. J Clin Virol.

